# Using Ecological Momentary Assessment to Measure Impulsivity among Trauma-Exposed Adults

**DOI:** 10.1007/s10862-025-10248-2

**Published:** 2025-09-13

**Authors:** Emily Heinlein, Cameron P. Pugach, Blair E. Wisco

**Affiliations:** 1 Department of Psychology, University of North Carolina at Greensboro, PO Box 26170, Greensboro, NC 27401, USA

**Keywords:** Posttraumatic stress disorder, PTSD, Risky behavior, Impulsivity, Ecological momentary assessment, Negative urgency

## Abstract

Impulsivity has historically been measured with retrospective self-reports rather than ecological momentary assessment (EMA). This paper assesses momentary engagement in risky behavior and whether it is driven by momentary negative affect/situations and positive affect/situations, reflecting the negative and positive urgency facets of impulsivity. The present study used EMA to analyze whether momentary negative and positive feelings and situations were associated with engagement in risky behavior. This study also investigates whether PTSD diagnostic status moderated these associations. The sample included 80 trauma-exposed community members (39 with PTSD, 41 without PTSD) who completed up to 17 surveys per day for three days (total number of completed surveys = 2,158). This analysis produced more evidence for negative urgency than positive urgency, with negative affect and situations both significantly predicting risky behavior at the within-subjects level. Negative urgency was either not moderated by PTSD or possibly tempered by PTSD. This study offers a novel approach for measuring momentary urgency within trauma-exposed samples using EMA. Although this secondary analysis was limited, it provides promising new avenues for research.

Impulsivity is a multifaceted and multidimensional concept that is an important transdiagnostic risk factor in psychopathology research (Billieux et al., 2021; Kleinman et al., 2012; Ortal et al., 2015). Posttraumatic stress disorder (PTSD) is one of many disorders that has been associated with engagement in impulsive behavior. PTSD is defined in the *Diagnostic and Statistical Manual of Mental Disorders-5* as a trauma- and stressor-related disorder that includes symptoms of trauma intrusions, avoidance, negative cognition/mood, and alterations in arousal following direct or indirect exposure to a traumatic event *(DSM-5*; American Psychiatric Association, 2013). Specific symptoms include several related to impulsivity, including marked changes in reactivity, irritability, angry outbursts, or reckless and self-destructive behaviors (*DSM-5*; American Psychiatric Association, 2013).

## PTSD and Risky Behavior

PTSD is also associated with a variety of other impulsive behaviors beyond those specified in the DSM-5 diagnostic criteria (e.g., substance use, risky sexual behaviors, sensation-seeking behaviors; Weiss et al., 2012, Weiss et al., 2015). Given the link between PTSD symptoms and engagement in risky behaviors (Contractor et al., 2017), researchers have examined what causes those who are experiencing more severe trauma-related symptoms to engage in more risky behavior. One model that explored impulsive behaviors within trauma-exposed samples was the compulsive re-exposure hypothesis, which hypothesized that individuals with PTSD are more likely to experience boredom or lack of stimulation after exposure to trauma and therefore seek out sensation-seeking activities to feel stimulated (Ardino, 2012; Joseph et al., 1997). Another framework proposed was the self-medication model, which proposed that individuals engage in impulsive behaviors to cope with their distress and PTSD symptoms (Chilcoat & Breslau, 1998). This study focuses on two specific facets of impulsivity that have been more recently identified as important factors when examining risky behavior in the context of PTSD: negative and positive urgency (Hahn et al., 2015; Morris et al., 2020; Mirhesham et al., 2017; Price et al., 2017; Roley et al., 2017; Weiss et al., 2015).

## PTSD and Urgency

Urgency can be defined as the tendency to engage in rash or risky behaviors in response to emotion. Initially, urgency was conceptualized exclusively as a response to distress, or negative emotions (Whiteside & Lynam, 2001). More recent evidence indicates that there are two types of urgency: negative urgency, defined as the tendency to engage in risky or impulsive behaviors in the presence of negative feelings or emotions (Weiss et al., 2015), and positive urgency, defined as the tendency to engage in impulsive behaviors in the presence of positive emotions (Cyders & Smith, 2008b; Weiss et al., 2015). Recent contributions to the field highlight the role of negative and positive urgency as drivers in the relationship between PTSD and impulsive behavior (Weiss et al., 2015). Trait-level urgency has been found to predict PTSD status in previous research (Morris et al., 2020). Although some researchers argue that urgency should be conceptualized as one construct, (rather than differentiating negative and positive urgency separately; Sperry et al., 2018) recent findings in this field suggest that measuring negative and positive urgency separately may be more appropriate (Sharpe et al., 2021).

Negative urgency has been identified in prior studies as both an important risk factor for developing PTSD and an exacerbating factor for PTSD symptoms (Mirhashem et al., 2017). Individuals with PTSD have been shown to report higher trait-level negative urgency than those without PTSD (James et al., 2014; Peck et al., 2022). Thus, negative urgency has emerged as a significant predictor of PTSD symptoms. In their study exploring the relationship between impulsivity and different PTSD symptom clusters among a trauma-exposed sample, Roley et al. (2017) found that negative urgency was the most predictive of -and was significantly associated with- all PTSD symptoms clusters when compared to three other facets of impulsivity measures by the UPPS (lack of perseverance, lack of premedication, sensation seeking). Negative urgency has also been found to mediate the association between PTSD symptoms and risky behaviors (Morris et al., 2020; Weiss et al., 2015).

Research on positive urgency has provided evidence for the role of positive affect as a driver of impulsive behavior. Although some studies show that positive urgency is associated with PTSD (Kaysen et al., 2014; Weiss et al., 2015; Zapowski et al., 2009), there is evidence that negative urgency is more strongly associated with PTSD than positive urgency. For example, in their study observing the interrelated constructs of PTSD, urgency, and substance related problems, Mirhashem et al. (2017) found that although positive urgency may have an important role within the pathway to substance related problems, the effect of positive urgency on PTSD symptoms was not well-founded. Price et al. (2017) found that in a sample of participants with comorbid PTSD and SUD, the association between positive urgency and PTSD was markedly weaker than the relationship between PTSD and negative urgency. These findings suggest that although both positive and negative urgency may be relevant to PTSD, negative urgency plays a more established role in PTSD symptom severity and maintenance. Mixed findings surrounding an association between PTSD and positive urgency could be explained by the dampening of positive emotions within individuals with PTSD. The DSM-5 recognizes “persistent inability to experience positive emotions” as a PTSD symptom, possibly suggesting a lesser role for positive than negative urgency in this diagnostic group (American Psychiatric Association, 2013).

## Benefits of Measuring Impulsivity with EMA

Impulsivity has historically been assessed with trait measures that rely on retrospection and self-awareness of why one engages in risky behavior. By contrast, daily life methods such as EMA allow for the direct assessment of urgency by testing whether emotional states contribute to engagement in risky behavior in real time, naturalistic contexts. More recently, EMA research has explored the state-level association between negative urgency and impulsive behaviors. A recent study conducted by Sharpe et al. (2021) explored a cascade model of urgency; their findings supported a model in which daily experiences of stress lead to affective shifts, which then drive impulsivity (Sharpe et al., 2021). Feil et al. (2020) found that individuals reported that they acted on impulse more when experiencing higher levels of negative affect than usual for them. EMA affords examination of how individual differences in psychopathology affect within-person facets of impulsivity (e.g., positive and negative urgency), whereas retrospective self-report is limited by analyzing these relationships only at the between-person level. These strengths explain the recent application of EMA to study impulsivity in daily life (e.g., Sperry et al., 2021). Few studies to date have assessed impulsivity using EMA in individuals with PTSD and have only assessed certain risky behavior variables related to substance use (Black et al., 2018; Lane et al., 2019).

## The Present Study

The present study is a secondary analysis that assesses momentary engagement in risky behavior, which is identified as our impulsive behavior variable. The goal of this analysis is to clarify the possible roles of negative and positive affect and situations in everyday engagement in risky behavior in trauma-exposed adults. We operationalized negative and positive urgency in two ways. First, as endorsement of negative or positive affect among participants and how that as associated with engagement in risky behavior; and the second, as endorsement of stressful or positive situations and how that was associated with engagement in risky behavior. Our analysis has the following hypotheses: (1) negative affect and negative situations will be positively associated with contemporaneous engagement in risky behavior, reflecting negative urgency, (2) positive affect and positive situations will also be positively associated with contemporaneous engagement in risky behavior, reflecting positive urgency, (3) PTSD status will moderate the associations between negative affect/situations and risky behavior, such that this association will be stronger for individuals diagnosed with PTSD. We also sought to examine (4) whether PTSD status will moderate the associations between positive affect/situations and risky behavior, but we consider our fourth hypothesis as more exploratory given the mixed results from prior research surrounding positive urgency and PTSD.

## Methods

### Participants

This is a secondary analysis of the Ambulatory Physiological Assessment of PTSD study dataset; the original study included 80 trauma-exposed community members. The average age of participants was 21.79 (*SD* = 4.21). Participants were primarily women (*n* = 60; 75.00%) and predominantly White (*n* = 28; 35.00%) or Black (*n* = 23; 28.75%). There were 39 participants that met criteria for PTSD; 41 participants did not meet criteria PTSD and were considered trauma exposed controls. Diagnoses were based on the Clinician Administered PTSD Scale for *DSM-5* (CAPS-5; Weathers et al., 2013b). Eligibility for the larger study required participants to be between the ages of 18 and 40, have a body mass index between 18.5 and 34.9, speak English, and report at least one DSM-5 Criterion A traumatic event that occurred more than one month prior to participation. Exclusion criteria included pregnancy, history of cardiovascular disease, trauma exposure in the past month, medications known to affect cardiovascular functioning, psychosis, or reporting dissociative symptoms on a prescreening questionnaire.

85 trauma-exposed community members were recruited into the study from a mid-sized city in the southeastern United States. Five participants were excluded from analyses for reporting an exclusion criterion after consent (*n* = 1) or not completing all study procedures (*n* = 4), leaving the final sample of 80 participants.

### Measures

#### PTSD Status

PTSD was measured using the Clinician-Administered PTSD Scale for DSM-5 (CAPS-5; Weathers et al., 2013b). The CAPS-5 is a structured clinical interview assessing the 20 core symptoms of PTSD. Items are rated on a 5-point Likert scale ranging from 0 (*Absent*) to 4 (*Extreme/Incapacitating*). Items are summed to yield an overall score of symptom severity. To determine diagnostic status, DSM-5 criteria were used, with symptoms rated “2” or higher counting towards diagnostic criteria. All CAPS-5 interviews were administered by trained graduate students. Interviews were audio recorded and later rated by a second-rater blind to diagnostic status; interrater reliability was excellent (ICC = 0.99).

### EMA Measures

All EMA items started with the stem “In the past ten minutes” and asked participants to provide their rating on the same 7-point Likert scale (1 = *Not at all* to 7 = *Very much*). Continuous scores were used for all analyses; for descriptive purposes, scores of “2” or higher were counted as endorsement of a particular item.

#### Risky Behavior

Risky behavior was measured with the single item “*I did something risky*.” Participants rated the extent to which they participated in risky behavior in the prior 10 min at each EMA timepoint.

#### Negative Affect and Situation

Negative affect was operationalized as the mean of 10 items: afraid, worried, horrified, helpless, numb, guilty, ashamed, angry, sad, and disgusted. Multilevel reliability values for the negative emotion composite, calculated using McDonald’s Omega, were ω_B_ = 0.78 and ω_W_ = 0.81. Stressful situation was measured with the single item “*my situation was stressful*.”

#### Positive Affect and Situation

Positive affect was operationalized with a single item *“I felt happy.”* Positive situation was also measured with the single item “*my situation was positive*.”

### Procedure

Recruitment methods for participants included community flyer postings and online advertisements; participants were also recruited from a psychology clinic, and from a sample of trauma-exposed adults who completed prior studies in the lab and consented be contacted about future studies. Participants who were interested first completed online prescreening questionnaires. The questionnaires asked about lifetime trauma history, PTSD symptoms experienced in the previous month, medication, psychosis, and health conditions known to affect physiological data (to measure inclusion/exclusion criteria). Participants with elevated PTSD symptoms on the prescreening questionnaire were oversampled to ensure relatively equal numbers of participants with and without PTSD, and recruitment was stratified to ensure matching based on trauma type, age, race and ethnicity, and body mass index.

Participants completed two lab sessions and three days of ambulatory assessment (i.e., EMA paired with ambulatory physiological monitoring). The first lab session required participants to provide informed consent and complete structured clinical interviews, which included the CAPS-5 interview. The three days of ambulatory assessment were completed within approximately one week of the first lab session. At the end of the first lab session, participants were connected to mobile physiological devices and oriented to the EMA questionnaires. Participants were provided with a tablet computer to take home to use for EMA questions and returned to the lab on each morning of ambulatory assessment to be connected to an ambulatory device and receive a new, freshly charged tablet for EMA. Participants completed additional self-report questionnaires, a script-driven imagery procedure, and were provided monetary compensation at the final lab session.

EMA prompts were delivered up to 17 times per day between 10 a.m. and 10 p.m. Prompts were delivered on a randomized time interval between 30 and 90 min (*M* = 53.67 min, *SD* = 13.34, range 31–73). Participants were alerted to complete a prompt by an alarm on the tablet. After the alarm notified the participant, they had 20 min to respond. At each signal prompt, participants were asked to complete the EMA survey based on their experience over the previous 10 min.

In the present study, only first lab session clinical interview (CAPS-5) and EMA question items about risky behavior, emotion, and positive/negative situations were analyzed.

### Data Analysis

Multilevel models were used for this analysis. Multilevel models were conducted in Mplus Version 8.10. Descriptive statistics were calculated using SPSS. Negative affect, positive affect, stressful situation, and positive situation were all person mean centered. The unconditional model was first estimated using risky behavior as the outcome. Four models were then estimated with the different indicators of experience (i.e., negative affect, stressful situation, positive affect, and positive situation) as the level 1 predictor, and PTSD status (0 = PTSD absent and 1 = PTSD present) as the level 2 moderator. We also included day of assessment (0, 1, or 2) as a level 1 covariate and age, sex (0 = Men, 1 = Women), and person mean of the relevant level 1 predictor as level 2 covariates. The following model was used for each level-one predictor:

Level 1:

$${\mathrm{RiskyBehavior}}_{ij}=\beta_{0j}+\beta_{1j}(\mathrm{Affect}\;\mathrm{or}\;\mathrm{Situation})+\beta_{2}(\mathrm{Day})+r_{ij}$$

Level 2:

$$\beta_{0j}=\gamma_{00}+\gamma_{01}(\mathrm{PTSD}\;\mathrm{Status})+\gamma_{02}(\mathrm{Sex})+\gamma_{03}(\mathrm{Age})+\;\gamma_{04}(\mathrm{Person}\;\mathrm{Mean}\;\mathrm{or}\;\mathrm{Affect}\;\mathrm{Simulation})+u_{0j}$$

$$\beta_{1j}=\gamma_{10}+\gamma_{11}(\mathrm{PTSD}\;\mathrm{Status})+u_{1j}$$

### Sensitivity Power Analysis

This study is a secondary analysis of a larger dataset that was collected for different aims; to assess that our hypotheses are properly powered, we ran a sensitivity analysis. The following sensitivity analysis was conducted in R (Version 4.3.1) using the lmer() and simr() packages for multilevel modeling. This sensitivity analysis was conducted using a smallest effect size of interest approach. This method analyzes the smallest possible effect sizes that the study is powered to find given the collected sample size. Analyses for each hypothesis were conducted with a target power of 80%, alpha of 0.05, and *n* = 80.

The results of our sensitivity analysis for our first hypothesis showed that the study was adequately powered to detect coefficients as small as 0.150 for negative affect, and 0.037 for negative situation as predictors of risky behavior (all coefficients are unstandardized). Results showed that our second hypothesis would be powered to detect coefficients as small as 0.034 for positive affect, and 0.032 for positive situation as predictors of risky behavior. Our third and fourth hypotheses, that PTSD status will moderate these associations, found that our study is adequately powered to detect moderations effects as small as 0.18 for negative affect, 0.047 for negative situation, 0.05 for positive affect, and 0.042 for positive situations.

## Results

### Daily Life Descriptive Characteristics

In total, 2,158 EMA surveys were completed by participants (trauma-exposed control group = 1,132, PTSD group = 1,026), with an average of 26.98 surveys per participant. Table 1 shows descriptive statistics, including intraclass correlation coefficients (ICCs) and between- and within-subjects correlations for key study variables. Of note, risky behavior was endorsed on 4.68% (*n* = 101) of EMA surveys and approximately 41.25% (*n* = 33) of participants endorsed risky behavior at least one time in the study. The ICC showed that the variance attributable to between-subjects effects for risky behavior was 0.303, indicating that ~ 70% of the variance in risky behavior was within-subjects.

Means-as-outcomes models were used to examine the influence of PTSD group status on daily life study variables. Descriptive statistics for each variable broken down by group are depicted in Table 1 and show that group status was a significant predictor of negative affect (β = 0.40, *SE* = 0.07, *p* =.00), positive affect (β= −1.23, *SE* = 0.25, *p* =.00). and positive situation (β= −0.61, *SE* = 0.21, *p* =.03), but not stressful situation (β = 0.26, *SE* = 0.17, *p* =.14). No differences were observed between groups for risky behavior (β = 0.02, *SE* = 0.07, *p* =.82).

### Negative Urgency and Moderating Effect of PTSD Status

Negative urgency was operationalized in two separate MLMs as the momentary within-subjects relationships between (1) negative affect and risky behavior and (2) perceived situational stress and risky behavior. Separate slopes-as-outcome models tested whether PTSD group status moderated these associations. Results are shown in Table 2. In the first model, the level 1 relationship between risky behavior and negative affect was significant (*b* = 0.44, 95% CI [0.114, 0.774], *SE* = 0.17, *p* =.008,); meaning, the more negative affect participants experienced relative to their typical level of negative affect, the more likely they were to endorse engaging in risky behavior. This association was qualified by a significant cross-level interaction effect of PTSD group status. In contrast to our hypothesis, people with PTSD showed weaker associations between negative affect and risky behavior, relative to trauma-exposed controls (*b* = −0.36, 95% CI [−0.01, 0.37], *SE* = 0.17, *p* =.038). To explore which specific negative emotions were associated with risky behavior, we ran supplemental analyses with the same model run for each negative emotion separately (see Table 4).

In the second model, the level 1 relationship between perceived stressful situations and risky behavior was significant (*b* = 0.10, 95% CI [0.021, 0.187] *SE* = 0.04, *p* =.014) however, the level 2 interaction was not; PTSD presence did not significantly moderate the relationship between perceived stressful situations and risky behavior (*b* = −0.04, 95% CI [−0.13, 0.06], *SE* = 0.05, *p* =.430).

### Positive Urgency and the Moderating Effect of PTSD

Positive urgency was operationalized in two more separate MLMs as the momentary within-subjects relationships between (1) positive affect and risky behavior and (2) perceived positive situations and risky behavior. Slopes-as-outcome models tested whether PTSD group status moderated these associations. Results are shown in Table 3. In the third model, the level 1 relationship between positive affect and risky behavior was not significant (*b* = −0.01, 95% CI [−0.03, 0.02], *SE* = 0.01, *p* =.585,). In the cross-level interaction, PTSD was not found to be a significant moderator in the relationship between positive affect and risky behavior (*b* = −0.01, 95% CI [−0.04, 0.02], *SE* = 0.02, *p* =.511).

In the fourth model, the level 1 relationship between perceived positive situation and risky behavior was not significant (*b* = 0.003, 95% CI [−0.04, 0.05], *SE* = 0.02, *p* =.895,). The cross-level interaction for positive situations was also not significant; PTSD presence did not significantly moderate the relationship between positive situations and risky behavior (*b* = −0.01 95% CI [−0.02, 0.04], *SE* = 0.03, *p* =.619) (Table 4).

## Discussion

A body of work that examines urgency in everyday life has recently been established (Feil et al., 2020; Sharpe et al., 2021; Sperry et al., 2021), however this study offers a novel approach for measuring momentary urgency within trauma-exposed samples. Although risky behavior was endorsed relatively infrequently among this sample, there was enough variation among reported severity of risky behavior and occurrence across the sample to justify the analysis. In line with our first hypothesis, we found that both negative affect and stressful situations were associated with greater engagement in risky behavior for the group. This analysis produced more evidence for negative urgency than for positive urgency, as neither positive affect nor positive situation was associated with risky behavior. In terms of PTSD status, urgency was either not moderated by PTSD status or possibly weakened by presence of PTSD. The one significant moderation finding was in the opposite direction as predicted and seems sensitive to measurement, as it only emerged for negative affect and did not replicate with continuous PTSD severity scores.

We found that both negative affect and negative situation were significant level 1 predictors of risky behavior, which aligns with previous research. Prior research has established an association between negative affect and risky behaviors at the between-person level using trait measures of these constructs. A few recent studies have explored effects of negative urgency with EMA in predominantly non-clinical samples (Feil et al., 2020; Sharpe et al., 2021; Sperry et al., 2021). Our findings expand upon this prior research and include within-persons effects in a trauma exposed sample with psychopathology. In other words, prior research has established that people who are higher in negative affect than the average person also engage in more risky behavior than the average person (Sperry et al., 2018). Prior studies have also indicated that the moments in which individuals feel worse than usual (or are in negative situations) are also the moments in which they engage in risky behavior. Our study builds upon this literature to support this finding in a clinical trauma-exposed sample. Clinically, these within-person effects may be more relevant because clinicians work with trauma-exposed individuals to identify personal triggers and vulnerability factors.

We also found no evidence of positive affect or positive situation having a role in engagement of risky behavior. Recent work has suggested that negative and positive urgency might reflect a single underlying construct and could be modeled together (Billieux et al., 2021; Cyders and Smith, 2008; Smith & Cyders, 2016). Our findings argue against collapsing these two types of urgency, as they had differing effects on risky behavior in our sample. Positive urgency is a more recent contribution to trait impulsivity research (Cyders et al., 2007) and its role in PTSD is not as well supported as negative urgency. Positive urgency may be more relevant for other disorders than for PTSD. For example, positive urgency has a similar association with alcohol use problems as negative urgency (Coskunpinar et al., 2013; Cyders et al. 2007). Future research should examine EMA measures of positive urgency in other samples that might be more likely to show evidence of positive urgency as a driver of risky behavior, such as bipolar disorder.

Participants with PTSD did not report more risky behavior or demonstrate more negative or positive urgency in the slopes-as-outcomes models than trauma-exposed controls, which was counter to our predictions. There has been some evidence in prior research to suggest that different contexts may affect the association between PTSD and risky behavior. One context that could affect engagement in risky behavior is trauma type; a study done by Augs-burger and Elbert (2017) showed that among a group of trauma exposed refugees, association between risk-taking behavior and exposure to traumatic events was predicted by the type of trauma experienced. Another factor that could affect engagement in risky behavior is subtypes of PTSD (i.e., internalizing vs. externalizing). Because PTSD is such a heterogeneous disorder, it may be that only a subset of those with PTSD (i.e., those with the externalizing subtype) will engage in risky behavior. Indeed, prior research indicates that externalizing, but not internalizing, symptoms mediate the association between childhood trauma and risky behavior during adolescence (Jones et al., 2013). Another important consideration within EMA studies that operationalize affect within urgency could be affect lability, or rapid shifts in emotions. Jones et al. (2021) found that affect lability moderates the associations of both positive and negative urgency with PTSD such that among individuals with high affect lability, negative and positive urgency were not associated with PTSD. It is possible that an association with PTSD might emerge in larger samples that are able to account for affect lability in their statistical models.

### Limitations and Future Directions

There were several limitations that should be taken into consideration within the present study. First, our sample was predominantly female, civilian, and had a limited age range; results may not generalize to men, military veterans, children, or older adults. Second, the EMA survey only included one item devoted to risky behavior and did not evaluate the type of risky behaviors among individuals who endorsed the item, which relied upon their subjective interpretation of the term. There are both advantages and disadvantages to measuring a construct, such as risky behavior, with a single item. Some advantages of measuring risky behavior this way include minimizing response fatigue and therefore reducing participant burden and increasing feasibility in EMA assessments when an assessment is administered multiple times a day. The term “risky behavior” also allows for generalizability across multiple kinds of risk. This addresses a gap in the literature where studies often specify a type of risky behavior (i.e. substance use). However, there are also several disadvantages to measuring risky behavior in a single item that must be considered. For example, single-item measurement lacks the reliability of multi-item scales and often cannot capture complexity within multidimensional constructs. Future studies may consider including trait measures of risky behavior or including experimental laboratory designs to measure risk taking (Bechara et al., 1994; Lejuez et al., 2002) to validate single item questions about risk in EMA protocols.

This study also did not include any established trait measures specifically dedicated to measuring negative and positive urgency. We recommend that future studies that use this method include trait measures such as the UPPS-P to validate EMA-assessed negative and positive urgency, though it should be noted that within-person measures of momentary urgency often fail to demonstrate relationships with dispositional measures of impulsivity (e.g., Sperry et al., 2021). Our sample also included a limited number of participants who reported engaging in risky behavior over the three days of EMA data collection, limiting statistical power to detect level 2 effects and cross-level interactions. Risky behavior was also endorsed relatively infrequently, which could account for the lack of differences observed between groups for risky behavior. Finally, ecological momentary assessment can place some unexpected burdens on the participant. For example, it should be acknowledged that EMA data collection can be onerous depending on the number of surveys each participant had to complete, and the requirement to report to the lab each day for ambulatory monitoring. Wearing the ambulatory monitors could also cause discomfort throughout the day, which could possibly affect the participants’ responses to questions about their mood or their engagement in risky behavior.

Despite these limitations, this secondary analysis provides an important proof of concept for this method of modeling negative urgency in trauma-exposed samples using EMA and lays a foundation for promising new avenues for research. For example, there is a growing body of work that identifies emotion dysregulation as a precursor to impulsive and risky behavior (Weiss et al., 2015; Gratz & Tull, 2010), however there is also a possible bidirectional relationship that could indicate that regular engagement in risky behaviors could lead to more emotional dysregulation. Future studies could include measures of emotion dysregulation as well as momentary affect and examine lagged effects to clarify the direction of the relationships between PTSD, emotional regulation, and impulsive behaviors, and could assess different facets of impulsivity besides urgency.

In general, our study found evidence for negative, but not positive, affect and situation as important drivers of risky behavior in a trauma-exposed community sample. Our findings add to a burgeoning line of research using this method to capture negative urgency with EMA and provides future research opportunities to examine additional within and between person moderators of this effect. We hope that future researchers will continue to use EMA to capture within-person fluctuations in both negative and positive urgency to increase our understanding of these important facets of impulsivity.

## Figures and Tables

**Table 1:** Descriptive statistics and bivariate correlations for key daily life variables

	Full Sample		TEC		PTSD		

Variable	*M*	*SD*	*M*	*SD*	*M*	*SD*	ICC
Negative Affect	1.33	0.54	1.18	0.34	1.50	0.66	0.43
Positive Affect	4.20	1.78	4.71	1.72	3.62	1.67	0.46
Perceived Situational Stress	1.89	1.44	1.77	1.29	1.97	1.55	0.27
Perceived Situational Positivity	4.17	1.49	4.36	1.85	3.92	2.00	0.43
Risky Behavior	1.10	0.57	1.10	0.57	1.10	0.56	0.30
	**Bivariate Correlation Matrix**						
	1		2		3	4	5
1. Negative Affect	-		− 0.418*		0.460*	− 0.253*	0.180*
2. Positive Affect	* − 0.546*		-		− 0.266*	0.577*	0.009
3. Perceived Situational Stress	0.402*		− 0.158		-	− 0.300*	0.173*
4. Perceived Situational Positivity	− 0.232*		0.610*		− 0.178	-	0.033
5. Risky Behavior	0.231*		0.074		0.193	0.064	-

The total sample (*N* = 80) was comprised of a trauma-exposed control group (TEC) of participants without current PTSD and a PTSD group. Descriptive statistics were calculated based on a total of 2,158 observations. *M* mean, *SD* standard deviation, and ICC intraclass correlation coefficient. Correlations below the diagonal depict between-subjects effects and correlations above the diagonal depict within-subjects effects

* *p* <.05

**Table 2:** Multilevel models examining momentary negative urgency

Negative Affect			
	*B* (*SE*)	*p*	95% CI
Intercept	1.127 (0.20)	< 0.001	[0.736, 1.518]
Negative Affect	0.444 (0.17)	0.008	[0.114, 0.774]
PTSD Status	−0.089 (0.06)	0.159	[−0.213, 0.035]
Negative Affect x PTSD Status	−0.360 (0.17)	0.038	[−0.701, −0.019]
Age	−0.001 (0.01)	0.951	[−0.019, 0.018]
Sex	0.117 (0.117)	0.318	[−0.013, 0.346]
Negative Affect Person Mean	0.266 (0.14)	0.065	[0.017 0.548]
Day	0.027 (0.02)	0.070	[0.002, 0.057]
Stressful Situation			
	*B* (*SE*)	*p*	95% CI
Intercept	1.021 (0.17)	< 0.001	[0.696, 1.348]
Negative Situation	0.10 (0.04)	0.014	[0.021, 0.187]
PTSD Status	−0.002 (0.08)	0.984	[−0.149, 0.146]
Negative Situation x PTSD Status	−0.038 (0.05)	0.430	[−0.133, 0.056]
Age	0.003 (0.01)	0.714	[−0.013, 0.019]
Sex	0.111 (0.13)	0.387	[−0.141, 0.346]
Negative Situation Person Mean	0.080 (0.06)	0.150	[−0.029, 0.188]
Day	0.025 (0.01)	0.041	[0.001, 0.049]

**Table 3:** Multilevel models examining momentary positive urgency

Positive Affect			
	*B* (*SE*)	*p*	95% CI
Intercept	1.051 (0.18)	< 0.001	[0.709 1.394]
Positive Affect	−0.006 (0.01)	0.585	[−0.029, 0.013]
PTSD Status	0.061 (0.10)	0.548	[−0.137, 0.258]
Positive Affect x PTSD Status	−0.010 (0.02)	0.511	[−0.041, 0.012]
Age	0.00 (0.01)	0.956	[−0.017, 0.016]
Sex	0.146 (0.14)	0.279	[−0.118, 0.411]
Positive Affect Person Mean	0.036 (0.03)	0.290	[−0.031, 0.010]
Day	0.029 (0.02)	0.188	[−0.014, 0.072]
Positive Situation			
	*B* (*SE*)	*p*	95% CI
Intercept	1.070 (0.18)	< 0.001	[0.726, 1.414]
Positive Situation	0.003 (0.02)	0.895	[−0.044, 0.050]
PTSD Status	0.029 (0.08)	0.725	[−0.131, 0.189]
Positive Situation x PTSD Status	−0.010 (0.02)	0.569	[−0.073, 0.044]
Age	−0.001 (0.01)	0.947	[−0.023, 0.016]
Sex	0.139 (0.13)	0.293	[−0.120, 0.398]
Positive Situation Person Mean	0.021 (0.03)	0.438	[−0.032, 0.074]
Day	0.030 (0.02)	0.090	[0.005, 0.064]

**Table 4:** Individual Negative Emotions’ Effects on Risky Behavior

Negative Emotion	B (SE)	95% CI

Afraid	0.430 (0.32)	[−0.196, 1.056]
Ashamed	0.229 (0.18)	[−0.118, 0.575]
Guilty	0.229 (0.14)	[−0.048, 0.507]
Disgusted	0.205 (0.13)	[−0.059,0.468]
Worried	0.145 (0.05)	[0.053, 0.236]
Helpless	0.094 (0.12)	[−0.135, 0.322]
Angry	0.060 (0.07)	[−0.080,0.201]
Sad	0.052 (0.08)	[−0.098, 0.201]
Numb	−0.036 (0.08)	[−0.193, 0.120]
Horrified	−0.043 (0.10)	[−0.233, 0.147]

Unstandardized parameter estimates for the coefficient of interest only are reported. Full models also included PTSD, Age, and Sex at Level 2 and Day at Level 1. Each emotion was rated on a Likert scale from 1 (not at all) to 7 (extremely)
